# *Schistosoma mansoni* and HIV acquisition in fishing communities of Lake Victoria, Uganda: a nested case–control study

**DOI:** 10.1111/tmi.12531

**Published:** 2015-05-27

**Authors:** Ali Ssetaala, Jessica Nakiyingi‐Miiro, Gershim Asiki, Nassim Kyakuwa, Juliet Mpendo, Govert J. Van Dam, Paul L. Corstjens, Pietro Pala, Leslie Nielsen, Jan De Bont, Giuseppe Pantaleo, Noah Kiwanuka, Pontiano Kaleebu, Anatoli Kamali, Alison M. Elliott

**Affiliations:** ^1^ UVRI‐IAVI HIV Vaccine ProgramEntebbeUganda; ^2^ MRC/UVRI Uganda Research Unit on AIDSEntebbeUganda; ^3^ London School of Hygiene & Tropical MedicineLondonUK; ^4^ Leiden University Medical CenterLeidenThe Netherlands; ^5^ International AIDS Vaccine InitiativeNew YorkNYUSA; ^6^ Centre Hospitalier Universitaire VaudoisLausanneSwitzerland; ^7^ Makerere College of Health SciencesSchool of Public HealthKampalaUganda; ^8^ Uganda Virus Research InstituteEntebbeUganda

**Keywords:** *Schistosoma mansoni*, circulating anodic antigen, HIV, susceptibility, fishing communities, Uganda, *Schistosoma mansoni*, antigène anodique circulant, VIH, sensibilité, communautés de pêcheurs, Ouganda

## Abstract

**Objective:**

It has been suggested that *Schistosoma mansoni*, which is endemic in African fishing communities, might increase susceptibility to human immunodeficiency virus (HIV) acquisition. If confirmed, this would be of great public health importance in these high HIV‐risk communities. This study was undertaken to determine whether *S. mansoni* infection is a risk factor for HIV infection among the fishing communities of Lake Victoria, Uganda. We conducted a matched case–control study, nested within a prospective HIV incidence cohort, including 50 HIV seroconverters (cases) and 150 controls during 2009‐2011.

**Methods:**

*S. mansoni* infection prior to HIV seroconversion was determined by measuring serum circulating anodic antigen (CAA) in stored serum. HIV testing was carried out using the Determine rapid test and infection confirmed by enzyme‐linked immunosorbent assays.

**Results:**

About 49% of cases and 52% of controls had *S. mansoni* infection prior to HIV seroconversion (or at the time of a similar study visit, for controls): odds ratio, adjusting for ethnicity, religion, marital status, education, occupation, frequency of alcohol consumption in previous 3 months, number of sexual partners while drunk, duration of stay in the community, and history of schistosomiasis treatment in the past 2 years was 1.23 (95% CI 0.3–5.7) *P* = 0.79. *S. mansoni* infections were chronic (with little change in status between enrolment and HIV seroconversion), and there was no difference in median CAA concentration between cases and controls.

**Conclusions:**

These results do not support the hypothesis that *S. mansoni* infection promotes HIV acquisition.

## Abstract (fr)

**Objectif:**

Il a été suggéré que *Schistosoma mansoni*, endémique dans les communautés de pêcheurs africains, pourrait augmenter la susceptibilité à l'acquisition du virus de l'immunodéficience humaine (VIH). La confirmation de cette hypothèse serait d'une grande importance pour la santé publique dans ces communautés à risque élevé pour le VIH. Cette étude a été entreprise pour déterminer si l'infection à *S. mansoni* est un facteur de risque pour l'infection VIH dans les communautés de pêcheurs du lac Victoria, en Ouganda. Nous avons mené une étude cas‐témoins appariés, imbriquée au sein d'une cohorte prospective d'incidence du VIH, comprenant 50 séroconversions VIH (cas) et 150 témoins, durant la période 2009‐2011.

**Méthodes:**

L'infection à *S. mansoni* avant la séroconversion VIH a été déterminée par la mesure de l'antigène anodique circulant (AAC) dans du sérum stocké. Le dépistage du VIH a été effectué en utilisant le test rapide ‘Determine’ et l'infection, confirmée par des méthodes ELISA.

**Résultats:**

49% des cas et 52% des témoins avaient une infection à *S. mansoni* avant la séroconversion VIH (ou au moment d'une visite d’étude similaire, pour les témoins); odds ratio (rapport de cotes), corrigé pour l'ethnicité, la religion, le statut matrimonial, l’éducation, la profession, la fréquence de la consommation d'alcool dans les trois mois précédents, le nombre de partenaires sexuels en état d’ébriété, la durée du séjour dans la communauté et l'historique de traitement de la schistosomiase au cours des deux dernières années = 1,23; IC95%: 0,3 à 5,7; P = 0,79. Les infections à *S. mansoni* étaient chroniques (avec peu de changement du statut entre l'inscription et la séroconversion VIH) et il n'y avait aucune différence dans la concentration médiane de l’AAC entre les cas et les témoins.

**Conclusions:**

Ces résultats ne soutiennent pas l'hypothèse que l'infection à *S. mansoni* favorise l'acquisition du VIH.

## Abstract (es)

**Objetivo:**

Se ha sugerido que *Schistosoma mansoni*, el cual es endémico en comunidades de pescadores Africanos, podría incrementar la susceptibilidad a la adquisición del virus de inmunodeficiencia humana (VIH). Si esto se confirmase, tendría una gran importancia para la salud pública de estas comunidades con alto riesgo de VIH. Este estudio se realizó para determinar si la infección por *S. mansoni* es un factor de riesgo para la infección por VIH en las comunidades de pescadores del Lago Victoria, Uganda. Hemos conducido un estudio de casos y controles apareados, anidado dentro de una cohorte prospectiva de incidencia del VIH, incluyendo 50 seroconversores por VIH (casos) y 150 controles, entre el 2009‐2011.

**Métodos:**

La infección por *S. mansoni* se determinó previamente a la seroconversión por VIH, determinando la presencia del antígeno circulante anódico (AAC) en suero, utilizando muestras de suero almacenadas. La prueba del VIH se realizó utilizando la prueba rápida Determine y la infección se confirmó mediante un ensayo de inmunoabsorción ligado a enzima.

**Resultados:**

El 49% de los casos y el 52% de los controles tenían un infección por *S. mansoni* antes de la seroconversión por VIH (o en el momento de una visita por un estudio similar para los controles): la razón de momios (OR), ajustando para etnia, religión, estatus marital, educación, ocupación, frecuencia de consumo de alcohol en los últimos tres meses, número de parejas sexuales mientras se está borracho, duración del tiempo que llevaban en la comunidad y el historial de tratamiento de la esquistosomiasis en los últimos dos años era de 1.23 (IC 95% 0.3‐5.7) P=0.79. Las infecciones por *S. mansoni* eran crónicas (con pocos cambios en el estatus entre el momento de incorporarse en el estudio y la seroconversión por VIH) y no había diferencia en la media de concentración del AAC entre los casos y los controles.

**Conclusiones:**

Estos resultados no apoyan la hipótesis de que la infección por *S. mansoni* promueve la adquisición del VIH.

## Introduction

Fishing communities in Uganda are disproportionately affected by HIV infection with incidence and prevalence four times the national average, or more 1, 2. These figures are attributed to high‐risk sexual behaviour and limited access to health services 1, 2. *Schistosoma mansoni,* a chronic, immunomodulating helminth infection, highly prevalent in fishing communities, may be an additional risk factor in these communities 3. It is estimated that over 16 million Ugandans are at risk of schistosomiasis and more than 4 million infected 4.

*S. haematobium* is a potential risk factor for HIV acquisition, because it directly affects the genital mucosa 5. *S. mansoni* infection has also been linked to increased risk of acquiring simian–human immunodeficiency virus (SHIV) and HIV in primate and human studies, respectively 3, 6, 7. Proposed systemic immunological mechanisms for this include up‐regulation of HIV co‐receptors and of Th2 T cells in which HIV may replicate more efficiently 3, 8. If confirmed, this would imply that control of *S. mansoni*, as well as of *S. haematobium*, could contribute to reducing the HIV reservoir among fishing communities and hence spread to the general population.

We conducted a matched case–control study, nested within a prospective HIV incidence study, to determine whether *S. mansoni* infection promotes HIV acquisition in Ugandan fishing communities.

## Methods

The study was conducted among five fishing communities on Lake Victoria, Uganda. This was a matched case–control study nested within a prospective HIV incidence cohort of 1000 individuals (males and females, aged 13–49 years) recruited in 2009, and followed for 18 months 2.

Within the prospective cohort, data on risk factors for HIV acquisition, and serum samples for HIV testing and storage, were obtained at enrolment and at six‐monthly follow‐up intervals. HIV testing was carried out using Determine rapid tests (Abbott Laboratories, Chicago, IL, USA), confirmed by enzyme‐linked immunosorbent assays (ELISA; Vironostika HIV Uni‐Form II plus 0 microelisa system, Biomerieux, Boxtel, Netherlands and Murex HIV‐1.2.0, Murex, Biotech Limited, Dartford, UK), with Western blot (Calypte Biomedical Corporation, Rockville, Maryland, USA) if ELISA results were indeterminate. During follow‐up, 59 individuals became HIV‐1 positive 2.

All cases available and willing to participate were selected. Fifty HIV seroconverters were included as cases, with 150 controls selected from those who remained HIV negative. For every case, three controls matched on age, sex and community of residence were randomly selected by computer using StataSE12 (1985–2011 StataCorp, College Station, TX, USA). Controls that were not willing to participate or could not be traced after more than three attempts were replaced.

At the case–control study visit, participants were interviewed regarding schistosomiasis risk factors. Stored serum was tested for *Schistosoma* circulating anodic antigen (CAA) at cohort enrolment, 6, 12 and 18 months using CAA‐specific up‐converting phosphor technology‐based lateral flow assay strips (UPT‐LF) 9, 10, and a CAA value ≥ 32 pg/ml was considered positive. Although CAA cannot distinguish *S. mansoni* from *S. haematobium*, the latter has not been found on the Ugandan shores of Lake Victoria 11, so a positive result was taken as indicative of *S. mansoni* infection. For cases, CAA serostatus was taken as the result for the last sample before HIV seroconversion. For controls, CAA serostatus was taken as the result for the sample corresponding to the last visit before HIV seroconversion for the matched case.

Stool samples were also collected during the case–control study visit (after the end of cohort follow‐up), for three consecutive days, to test for *S. mansoni* infection. Two laboratories participated. Microscopy was performed using the Kato–Katz method in one location, formol–ether concentration in the other location. Intensity of infection was defined based on WHO criteria as light (<100 eggs per gram (epg), moderate (100–399 epg) and heavy (≥400 epg).

Participants diagnosed with HIV infection (during follow‐up) were referred to The AIDS Support Organization (TASO) and Entebbe Hospital for care and treatment according to the Uganda Ministry of Health guidelines. Those diagnosed with *S. mansoni* infection (after the end of follow‐up) were treated with single‐dose praziquantel, 40 mg/kg. Participants diagnosed with other helminth infections were treated with a single dose of albendazole 400 mg.

Data were double‐entered using MS Access 2003 (1992–2003 Microsoft Corporation, Redmond, Washington, USA) and analysed using StataSE12.

The outcome was HIV seroconversion during follow‐up. The aim was to determine whether *S. mansoni* infection (assessed by serum CAA) was associated with the outcome (HIV seroconversion during follow‐up). Associations between stool microscopy results and serum CAA were also investigated.

Baseline age, sex and community of residence were matching factors. Matched conditional logistic regression was used to investigate univariable associations between HIV seroconversion and other demographic factors (marital status, education status, occupation, religion and behavioural risk factors; being away from home for at least two nights in the previous month; frequency of alcohol use; and number of sexual partners while under the influence of alcohol or drugs during the previous 3 months) and to investigate risk factors for *S. mansoni* infection (duration of stay in fishing community and history of treatment for schistosomiasis in the preceding 2 years). Covariates considered *a priori* to be potential confounders (marital status, religion and ethnicity) and those associated with *S. mansoni* at *P* ≤ 0.1 were assessed for confounding by adding them individually to the multivariable model and tested for goodness of fit using log likelihood ratio test. If the *S. mansoni*–HIV effect measure changed by 10% or more, the covariate was retained in the final model.

The study was approved by Uganda Virus Research Institute Science and Ethics Committee (FWA number 00001354) and the Uganda National Council of Science and Technology (FWA number 00001293). Participants aged 18 years and above were enrolled after giving written informed consent. Those aged 13–17 years were enrolled after written assent, with consent from their parents or guardians.

## Results

### Socio‐demographic characteristics

Characteristics of the cohort have been described 2. Among the 200 participants enrolled into this nested study, median age was 25 years (IQR 22–30 years). The highest proportion was males (56%), Roman Catholics (41%), married (63%), with incomplete primary‐level education (41%) and worked in fishing‐related jobs (66%).

### Schistosomiasis

The majority of participants (76%) had not received *S. mansoni* (praziquantel) treatment in the previous 2 years. Determination of *S. mansoni* infection status was based on serum CAA.

Half (51%) had *S. mansoni* infection at cohort enrolment, 48%, 52% and 52% at 6, 12 and 18 months, respectively. There was 96% agreement between the CAA status at enrolment and CAA status at the pre‐seroconversion (or equivalent control) time point (*κ*=0.91, *P* < 0.001). Compared to the stool results, serum CAA had a sensitivity of 84%, specificity 92% and a positive predictive value of 94%. Intensity of *S. mansoni* infection was assessed in 79 samples examined by the Kato–Katz method. Of those infected, 61% had light, 29% moderate and 10% heavy infections.

*S. mansoni* prevalence was highest in the 25–29 years age group, [66%, *P* < 0.001], higher among males than females [75% *vs*. 21%, *P* < 0.001] and increased with declining education level [*P* = 0.035]. Almost all participants who were engaged in fishing had *S. mansoni* infection [54/58, 93%; *P* < 0.001 compared to other occupational groups]. Being away from home frequently [*P* = 0.009] and long‐term residence in the fishing community [*P* = 0.012] were associated with *S. mansoni* infection.

### Association between *S. mansoni* infection and HIV acquisition

There was no difference between cases who acquired HIV infection, and controls who had not, in *S. mansoni* infection status based on CAA results at the preceding visit: 49% of cases and 52% of controls had *S. mansoni* infection [OR 0.83 (95% CI 0.39–1.77) *P* = 0.63]. The result was similar after adjusting for ethnicity, religion, marital status, education, occupation, frequency of alcohol consumption in previous 3 months, number of sexual partners while drunk, duration of stay in the community and history of schistosomiasis treatment (praziquantel) in the past 2 years [aOR 1.23 (95% CI 0.3–5.7) *P* = 0.79]. There was also no difference in median CAA concentration between cases and controls: 2906 and 6530 pg/ml, respectively (*P* = 0.16).

Factors associated with HIV acquisition in the case–control study were similar to those previously reported for the whole cohort: occupation, religion, frequency of alcohol consumption (2); Table 1. In addition, not having taken praziquantel treatment for *Schistosomiasis* in the last 2 years was associated with HIV acquisition [OR 8.25 (95% CI 1.3–51.1) *P* = 0.03].

**Table 1:** Multivariable conditional regression analysis of factors associated with HIV infection

Variable	Unadjusted		Adjusted		
	OR	95% CI	OR	95% CI	*P*‐value
Serum CAA	0.8	0.4–1.8	1.23	0.3–5.7	0.79
Marital status					
Premarital	1 Ref		1 Ref		0.5
Married Monogamous	1.00	0.4–2.6	1.14	0.19–6.76	
Married Polygamous	0.55	0.2–2.0	0.36	0.33–4.02	
Divorced/Widowed	1.36	0.4–4.4	0.52	0.55‐4.86	
Education Status					
Post‐primary	1 Ref		1 Ref		0.94
Completed Primary	1.42	0.6–3.6	0.64	0.1–3.9	
Incomplete Primary	1.20	0.5–2.8	0.94	0.2–5.2	
None	1.61	0.5–5.3	1.00	0.1–8.5	
Occupation					
Fishing	1 Ref		1 Ref		<0.005
Fishing related	1.13	0.4–3.1	13.54	1.8–101.3	
Bar/Hotel/restaurant	2.92	0.6–15.1	41.53	1.6–1071.3	
Business/trade	0.49	0.1–2.6	0.32	0.0–6.3	
Farming	1.57	0.4–6.0	8.84	0.7–110.6	
Other jobs	2.22	0.5–10.5	4.00	0.3–52.5	
Not earning	1.85	–	4.78	–	
Religion					
Catholic	1 Ref		1 Ref		0.018
Anglican	5.56	2.3–13.5	7.98	1.8–35.4	
Muslim	1.65	0.7–3.9	2.73	0.7–10.9	
Other	1.70	0.4–7.2	9.06	0.7–112.3	
Ethnicity					
Non‐Muganda	1 Ref		1 Ref		0.32
Muganda	0.8	0.4–1.6	0.50	0.1–2.0	
Alcohol use					
Never	1 Ref		1 Ref		0.013
Rarely	1.92	0.8–4.8	9.21	1.4–61.8	
Regularly	3.49	1.6–7.8	6.85	1.3–37.4	
Duration of stay					
5–45 years	1 Ref		1 Ref		<0.005
<5 years	3.77	1.6–8.6	7.56	1.5–37.6	
Bilharzia treatment in last 2 years					
Yes	1 Ref		1 Ref		0.034
No	2.49	1.0–6.3	8.25	1.3–51.1	
Sexual partners while drunk(pre‐infection)					
0	1 Ref		1 Ref		0.98
1	1.96	0.8–4.7	0.67	0.1–3.2	
≥ 2	1.58	0.4–6.0	0.48	0.0–7.0	

### Discussion and conclusion

We found that *S*. *mansoni* infection was not associated with HIV acquisition among adults in Lake Victoria fishing villages. As opposed to *S. haematobium*,* S. mansoni* is unlikely to cause genital lesions. This result therefore suggests that systemic immune modulation by *S. mansoni* does not significantly increase susceptibility to HIV acquisition.

By contrast, an experiment in macaques showed increased susceptibility to a simian–human immunodeficiency virus (SHIV) infection in *S. mansoni‐*infected animals 6. However, the macaque schistosome infections were recent at the time of SHIV exposure (7 weeks), egg counts were equivalent to moderate to heavy human infections, and the virus was administered rectally, encountering tissues directly affected by *S. mansoni* egg‐induced lesions. By contrast, our human participants had chronic *S. mansoni* infections (few had recently been treated, and status had not changed between enrolment and the pre‐seroconversion visit), many had light infections, and HIV infection would usually have occurred via the genital mucosa. Indeed, in a second macaque study, *S. mansoni* infection did not increase susceptibility when SHIV was administered intravenously, implying that route of infection was critical 7.

Cross‐sectional studies have provided conflicting evidence regarding HIV–*S. mansoni* associations. A survey focussing on women on the Tanzanian shores of Lake Victoria using serum CAA to determine *Schistosoma* infection found a positive association with HIV infection, and higher CAA levels among HIV‐positive women 12. However, the number of HIV‐infected women was small (21 of 345) so confidence intervals were wide, the prevalence of HIV infection was approximately 4.5 times lower than in the Ugandan fishing villages, and the catchment area extended to 25 km from the lake, so that a wide array of potential confounding factors in terms of environment and lifestyle may have come into play. A low prevalence of *S. haematobium* (3%) might also have contributed.

The cross‐sectional design of the Tanzanian study means that the sequence in which the infections occurred is unknown. An earlier study showed no positive association between *S. mansoni* and HIV infection and highlighted differences in occupation and origin of members of the population affected by the two infections 13.

Given the lack of association we observed between *S. mansoni* infection and HIV acquisition, we were surprised to observe a strong association between HIV infection and lack of praziquantel treatment in the last 2 years. It is difficult to draw robust conclusions from self‐reported behaviour. We presume this may be a marker for poor health‐seeking behaviour, rather than for a biological process. Participants were not examined or treated for *S. mansoni* by the research team during the cohort study.

Strengths of our study were prospective sample collection, recruitment restricted to a relatively uniform (fishing village) community, and matching and adjustment for potential confounders. The CAA assay allowed assessment of *S. mansoni* infection status at enrolment and immediately prior to HIV seroconversion, demonstrating chronicity of infection, and avoided potential concerns about differential egg excretion among HIV‐positive and HIV‐negative people 14. Although it can be difficult to assess causality in case–control studies, the prospective design of our cohort did allow us to assess *Schistosomiasis* status prior to HIV acquisition – an important improvement over studies with a cross‐sectional design.

While there are many potential benefits of *S. mansoni* control in heavily infected communities, our results suggest that an impact on HIV transmission is not one of them.
